# Female Hydrocele: A Rare Case of a Cyst of the Canal of Nuck in an Adult Woman

**DOI:** 10.7759/cureus.107694

**Published:** 2026-04-25

**Authors:** Carlos E Roas Ruiz, Rafael A Guzman, Ahmed Madan, Feras Othman, Adesola A Ogunsakin, Jaime T Lee Young, Mohammad Masri

**Affiliations:** 1 General Surgery, Larkin Community Hospital, South Miami, USA; 2 Oncology, Larkin Community Hospital, South Miami, USA; 3 Surgery, Larkin Community Hospital, South Miami, USA

**Keywords:** canal of nuck, case report, female hydrocele, groin mass, groin mass in female, groin swelling, hydrocele of canal of nuck, inguinal mass

## Abstract

The cyst of the canal of Nuck, also known as a female hydrocele, is an uncommon anomaly caused by the incomplete obliteration of the processus vaginalis in females during fetal development. Because its presentation is nonspecific and resembles other groin pathologies, it is frequently misdiagnosed. We report the case of a 33-year-old woman with a gradually enlarging, mildly painful swelling in the left groin. Imaging revealed a well-defined fluid-filled structure located within the left inguinal canal, which was removed through an open anterior approach, as it provides direct anatomical exposure of the cyst and surrounding inguinal structures. Intraoperatively, a bluish, fluid-filled cyst attached to the round ligament was identified. Histopathological analysis confirmed a cyst of the canal of Nuck lined by mesothelial cells. The postoperative recovery was uneventful, with no recurrence during follow-up. This report underscores the need to include this rare lesion in the differential diagnosis of female groin swellings. Ultrasound is the first-line imaging method; CT scan and MRI are also helpful for the preoperative assessment, facilitating accurate diagnosis and optimal surgical planning. Open anterior excision is the definitive, safe, and effective surgical approach for canal of Nuck cysts, offering excellent outcomes and minimal risk of recurrence.

## Introduction

A cyst involving the canal of Nuck is an uncommon finding in adult women, with only a few documented cases [1]. During fetal development, the canal of Nuck forms as a small peritoneal projection that follows the round ligament through the inguinal canal to the labia majora [2]. It represents the female equivalent of the processus vaginalis in males, which normally closes shortly after birth [3]. When closure fails, the tract may remain open, allowing fluid accumulation and formation of a cystic cavity known as a female hydrocele [4].

Although typically encountered in children, with a prevalence of approximately 0.76% in girls aged 0-16 years [5], it can also occur in adult women. Due to its rarity and nonspecific clinical presentation, it is frequently misdiagnosed as more common inguinal pathologies, including hernias, lymph node enlargement, lipomas, or Bartholin cyst [6-8]. Ultrasound, CT scan, and MRI can help delineate the lesion’s structure and relationship to surrounding tissues, occasionally revealing entrapped omental or adnexal components [7,8]. Recognizing these features before surgery can help the surgeon determine the best approach for definitive treatment.

## Case presentation

A 33-year-old female with non-relevant past medical history and surgical history of cyst excision from her left hand was evaluated for a soft-tissue mass in the left groin, present for five years, describing it as a mobile, mildly tender swelling that fluctuated in size, with no apparent association with physical activity or changes in position, and caused discomfort when direct pressure was applied. She denied fever, nausea, vomiting, or any bowel or urinary changes.

On examination, a freely mobile, slightly tender, well-circumscribed, non-erythematous mass measuring approximately 4 x 3 cm was palpated in the left inguinal region, with no change during the Valsalva maneuver and no overlying skin alterations. Her vital signs were stable (Table 1). She was not taking any regular medications and had no known drug allergies. Laboratory studies were notable for mild leukopenia without clinical significance (Table 2).

**Table 1:** Vital signs.

Vital signs	Values
Heart rate	67 bpm
Blood pressure	119/82 mmHg
Respiratory rate	14 rpm
Temperature	98.2°F
Oxygen saturation	100%

**Table 2:** Laboratory results. WBC: white blood cells; BUN: blood urea nitrogen; AST: aspartate aminotransferase; ALT: alanine aminotransferase

Labs	Patient result	Normal range
WBC	4.86 x 10^3/µL	5.87-11.57 x 10^3/µL
Neutrophil differential	57.40%	34.0-71.0%
Hemoglobin	13.7 g/dL	11.7-16.0 g/dL
Hematocrit	40.10%	36.0-47.7%
Platelet count	231.0 x 10^3/µL	150-450 x 10^3/µL
BUN	7 mg/dL	7-20 mg/dL
Serum creatinine	0.46 mg/dL	0.52-1.04 mg/dL
Total bilirubin	0.4	0.2-1.2
AST	26 U/L	14-41 U/L
ALT	15 U/L	7-33 U/L
Alkaline phosphatase	74 U/L	43-107 U/L

A CT scan of the left groin revealed a nodular cystic density involving the left lower anterior pelvic wall in the suprapubic region measuring 19×12 mm, of uncertain etiology. The discrepancy between the cyst size on imaging and the clinical findings may be explained by partial decompression of the cyst during the imaging study, likely related to its communicating nature with the peritoneal cavity, leading to underestimation of its true size.

**Figure 1:**
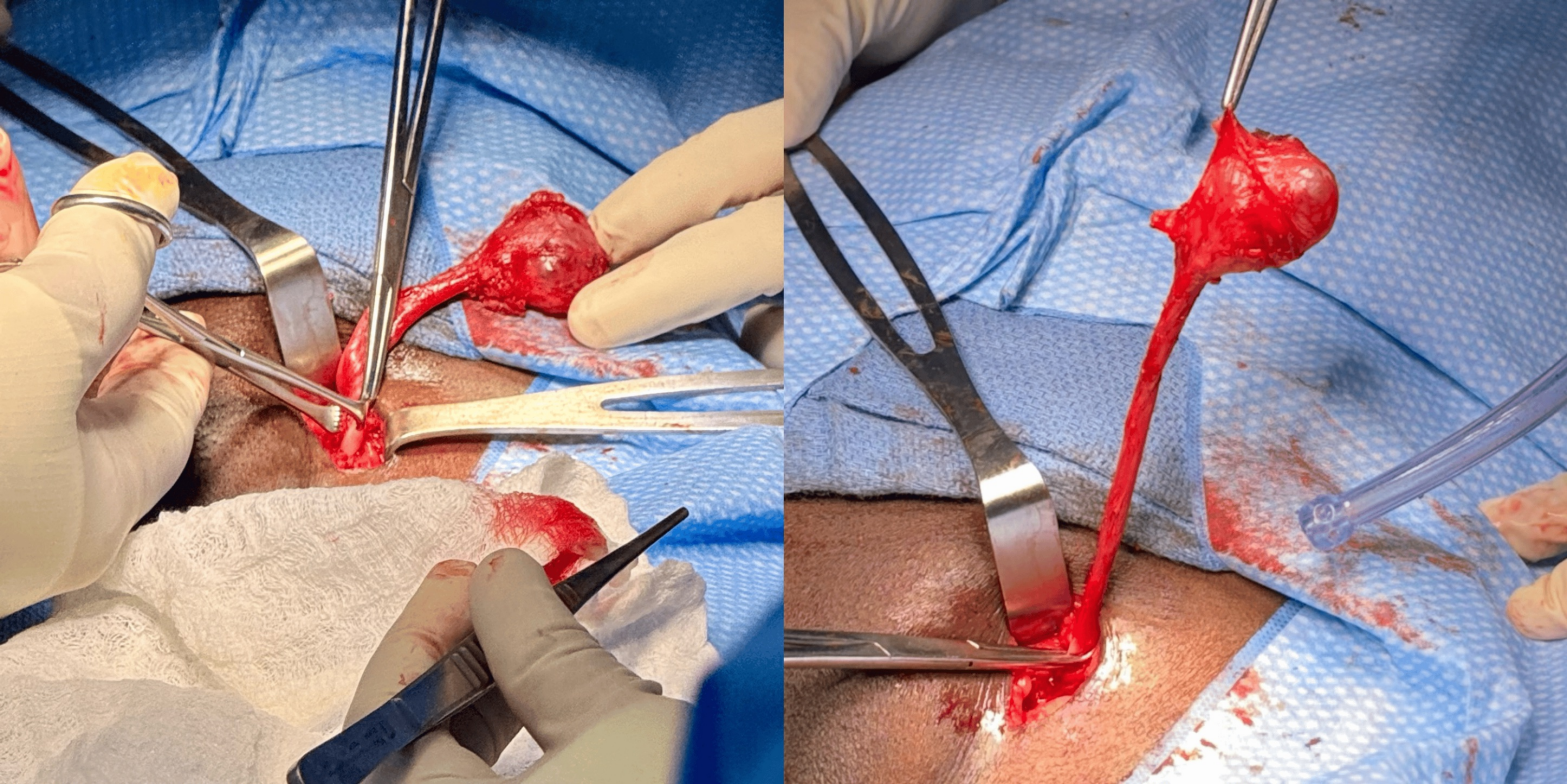
Cystic mass found, attached to an elongated pedicle that extended intraperitoneally.

The patient was taken to the operating room for an open anterior excision of the left inguinal mass. A 4 × 4 cm cystic mass was found, bluish in color and attached to an elongated pedicle that extended intraperitoneally. The pedicle was tied, excised, and the specimen was sent for pathological analysis (Figure 1). The inguinal canal defect was primarily repaired without mesh placement.

**Figure 2:**
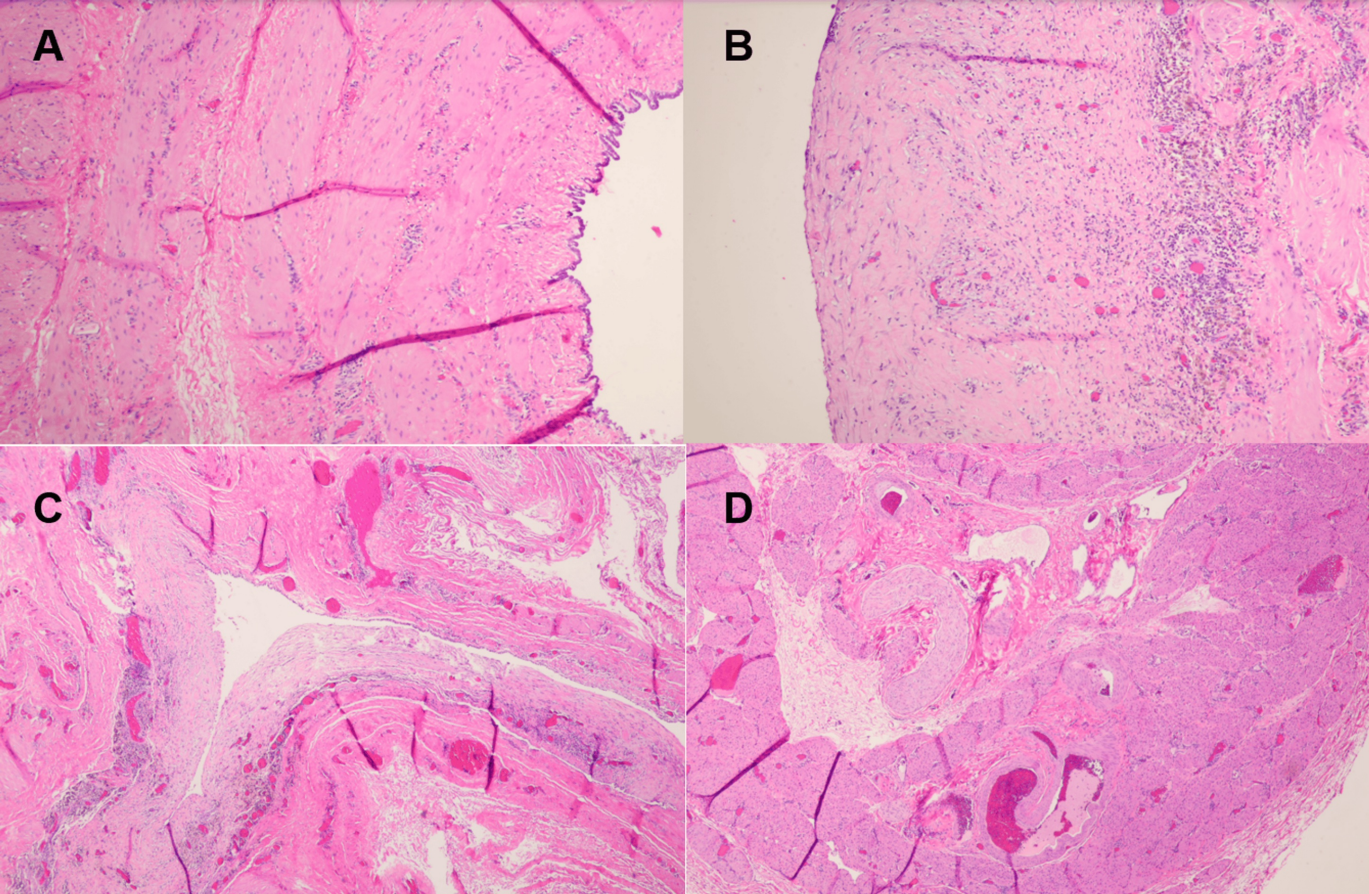
Histological slides obtained from the resected cyst. (A) Section of the cyst wall composed of dense fibrous connective tissue with interspersed smooth-muscle fibers and an inner lining of mesothelial-type cells, consistent with a cyst of the canal of Nuck (H&E, 100x). (B) Section of the cyst wall exhibiting focal chronic inflammatory infiltrates with small lymphocytes and scattered vascular channels within the fibromuscular stroma (H&E, 100x). (C) Cystic cavity bordered by a thick fibromuscular wall and lined by a single layer of flattened mesothelial-like epithelium; no evidence of atypia or malignancy (H&E, ×100). (D) Low-power view of the cystic wall and surrounding skeletal muscle fibers, confirming attachment to the distal round ligament (H&E, 40x).

The pathology report described a cystic structure lined by flattened to cuboidal mesothelial-like cells supported by a fibromuscular wall. Adjacent to the cyst, there was a nodular structure composed peripherally of smooth muscle and centrally of skeletal muscle fibers with intervening vascular channels. The overall morphologic and anatomic features were compatible with a cyst (hydrocele) of the canal of Nuck with an attached portion of the distal round ligament. No ovary or fallopian tube was identified, and the specimen was negative for atypia or malignancy (Figure 2).

The postoperative course was unremarkable. The patient was discharged the following day and remained asymptomatic, with no evidence of recurrence on clinical examination at six months of follow-up.

## Discussion

Named after Anton Nuck, a Dutch anatomist who first described this structure in 1691 [4], the canal of Nuck forms during the 12th week of embryological development and represents an outpouching of the parietal peritoneum [9]. Abnormalities of the canal of Nuck arise when the processus vaginalis remains patent in females, allowing fluid accumulation that can form a cyst or herniate internal organs, most commonly the bowel and ovary, though cases involving the uterus, bladder, fallopian tubes, and even both adnexa have been reported [9].

We present a rare case of a type II B cyst of the canal of Nuck (female hydrocele) in an adult woman, with only 400 cases documented worldwide, approximately [10], the vast majority occurring in the pediatric population, which makes this presentation uncommon.

Classification of the canal of Nuck cysts

Cysts of the canal of Nuck can be described according to the Counseller and Black classification (1941) as follows:

Type I (Non-communicating)

Type I does not communicate with the peritoneal cavity; it is the most common type, and the cyst may be located anywhere between the deep inguinal ring and the labia majora. These lesions typically present as stable, non-reducible inguinal masses without variation in size, helping differentiate them from hernias [3].

Type II (Communicating)

Type II maintains a patent connection with the peritoneal cavity and presents similarly to a communicating hydrocele in males, with fluctuation in size over time, as observed in our patient. Despite the absence of significant change with the Valsalva maneuver, this may be explained by a narrow or "pinhole" communication that limits immediate transmission of intra-abdominal pressure while still allowing gradual fluid exchange [11,12]. This was supported intraoperatively by the identification of a patent pedicle.

Type III (Hourglass)

Type III occurs when the cyst is constricted by the deep inguinal ring, resulting in both communicating and non-communicating components, and may clinically mimic a complex or incarcerated inguinal hernia [4].

More modern classifications, as presented in 2021 by Wang et al. [13], classify the canal of Nuck cysts by anatomical location: Type A: located subcutaneously over the inguinal canal; Type B: located in the inguinal canal; Type C: limited to the internal inguinal ring; Type D: spreads from the internal inguinal ring to the inguinal canal or subcutaneously.

Histologically, cysts of the canal of Nuck reflect their peritoneal origin. They are composed of a fibrous outer wall containing blood vessels and smooth muscle fibers, lined internally by a single layer of flattened to cuboidal mesothelial cells [14].

Clinically, this lesion usually appears as a soft, mobile, and generally painless mass in the groin or labia that does not vary with the Valsalva maneuver [15]. The size may vary depending on whether it communicates with the peritoneal cavity. Because it is a fluid-filled cystic lesion, it can transilluminate. In rare cases, when abdominal or pelvic viscera become entrapped, it may present as a strangulated indirect hernia with vascular compromise [16], constituting a surgical emergency.

The differential diagnosis for inguinal masses in females includes Bartholin’s cyst, lymphadenopathy, indirect inguinal hernia, post-traumatic hematoma, and both benign and malignant tumors, such as lipoma, vascular aneurysm, sarcoma, and, more rarely, cystic lymphangioma, leiomyoma, or epidermal cyst [17]. A hydrocele of the canal of Nuck is often mistaken for an inguinal hernia due to its rarity, limited clinician awareness, and the scarcity of information in surgical textbooks. Moreover, approximately one-third of cases are associated with an inguinal hernia, and most reported instances have been identified intraoperatively during surgery initially performed for a presumed hernia [14].

Ultrasound remains the first-line imaging method owing to its accessibility and diagnostic accuracy for this type of cystic lesion. It typically reveals a well-defined, hypoechoic or anechoic, sausage- or comma-shaped mass lying superficially and medial to the pubic bone in the inguinal canal, showing posterior acoustic enhancement and occasionally internal septations [18]. When sonographic findings are inconclusive, CT or MRI may be used.

On CT, a well-defined, low-density collection corresponding to fluid attenuation can be identified [19]. At the same time, MRI shows a thin-walled, sausage-shaped cystic structure with high signal intensity on T2 and low signal on T1-weighted sequences [18]. These techniques help delineate the anatomical characteristics of the cyst, communication with the peritoneal cavity, the extension into the inguinal canal [16], and assist in planning the optimal surgical approach.

Some authors have proposed that ultrasound-guided cyst aspiration can be considered for temporary symptomatic relief or for patients at high surgical risk, though this method is not definitive and carries a high recurrence rate [1]. The definitive treatment is hydrocelectomy [20]. Although there is no standardized surgical technique, the approach should be adapted to the cyst type, anatomical features, and the surgeon’s experience [4].

The anterior open approach is the method of choice and is the approach consistently reported across all published literature on this condition [4,13]. It provides direct anatomical exposure of the cyst and surrounding inguinal structures, requires only minimal dissection of skin, subcutaneous tissue, and the fascia of the external oblique muscle, and allows secure ligation of any peritoneal pedicle under complete direct visual control. In experienced hands, the procedure is suitable for same-day ambulatory surgery, postoperative pain is minimal and manageable with non-opioid analgesics alone, and a 3-4 cm skin incision closed with an intradermal suture heals with an excellent cosmetic outcome. If an inguinal ring defect is identified intraoperatively, it may be repaired using the patient's own tissue without prosthetic mesh, eliminating the long-term risks associated with foreign material. Closure of the inguinal defect with mesh may be reserved for cases where tissue quality precludes a reliable autologous repair [19].

Following complete surgical excision, the prognosis is favorable, and recurrence is exceedingly uncommon [21]. Postoperative complications are uncommon and generally minor and self-limiting, such as seroma formation or superficial wound separation. Long-term follow-up is typically unnecessary unless the patient develops new or recurrent symptoms [21].

## Conclusions

The cyst of the canal of Nuck is a rare condition in adult women that often mimics more common inguinal pathologies, leading to diagnostic delays or misinterpretation. Awareness of this rare entity is essential for accurate diagnosis and proper management. Imaging modalities, particularly ultrasound and MRI, are fundamental for preoperative assessment, allowing precise characterization of the cyst, evaluation of its relationship with adjacent structures, and differentiation from hernias or other soft-tissue lesions, findings that help guide the surgical approach. The anterior open approach is the safe, evidence-supported, and reproducible standard for surgical management of canal of Nuck cysts, consistent with the principle of primum non nocere: it provides direct anatomical access, complete visual control, minimal tissue trauma, excellent cosmetic results when the wound is closed with an intradermal suture, and with a favorable postoperative outcome.
